# A Plethora of Functions Condensed into Tiny Phospholipids: The Story of PI4P and PI(4,5)P_2_

**DOI:** 10.3390/cells12101411

**Published:** 2023-05-17

**Authors:** Ana Bura, Sara Čabrijan, Iris Đurić, Tea Bruketa, Antonija Jurak Begonja

**Affiliations:** Laboratory of Hematopoiesis, Department of Biotechnology, University of Rijeka, R. Matejcic 2, 51000 Rijeka, Croatia

**Keywords:** phosphatidylinositol-4-monophosphate, phosphatidylinositol-4,5-bisphosphate, trafficking, Golgi apparatus, plasma membrane, PI4K, PI4P5KI

## Abstract

Phosphoinositides (PIs) are small, phosphorylated lipids that serve many functions in the cell. They regulate endo- and exocytosis, vesicular trafficking, actin reorganization, and cell mobility, and they act as signaling molecules. The most abundant PIs in the cell are phosphatidylinositol-4-monophosphate (PI4P) and phosphatidylinositol-4,5-bisphosphate [PI(4,5)P_2_]. PI4P is mostly localized at the Golgi apparatus where it regulates the anterograde trafficking from the Golgi apparatus to the plasma membrane (PM), but it also localizes at the PM. On the other hand, the main localization site of PI(4,5)P_2_ is the PM where it regulates the formation of endocytic vesicles. The levels of PIs are regulated by many kinases and phosphatases. Four main kinases phosphorylate the precursor molecule phosphatidylinositol into PI4P, divided into two classes (PI4KIIα, PI4KIIβ, PI4KIIIα, and PI4KIIIβ), and three main kinases phosphorylate PI4P to form PI(4,5)P_2_ (PI4P5KIα, PI4P5KIβ, and PI4P5KIγ). In this review, we discuss the localization and function of the kinases that produce PI4P and PI(4,5)P_2_, as well as the localization and function of their product molecules with an overview of tools for the detection of these PIs.

## 1. Introduction

**Figure 1:**
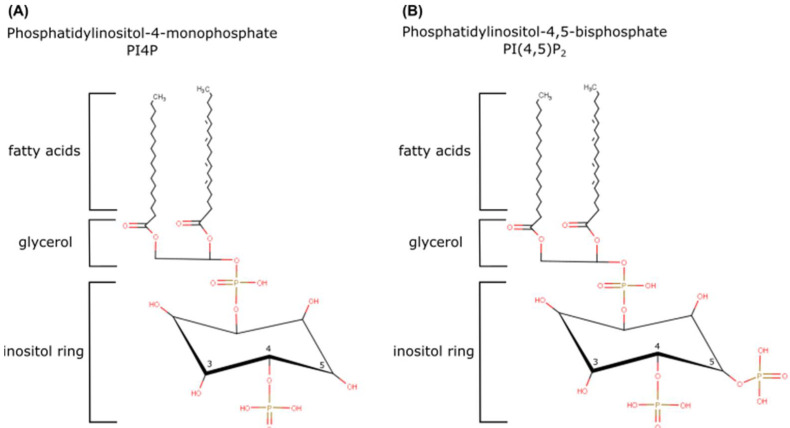
The structure of PI4P and PI(4,5)P_2_. Phosphoinositides can be phosphorylated on positions three, four, and five. They consist of an inositol ring, glycerol, and fatty acids. (**A**) PI4P is phosphorylated on position four of the inositol ring. (**B**) PI(4,5)P_2_ is phosphorylated on positions four and five of the inositol ring.

Phospholipids are abundant, complex, and highly diverse components of cell architecture [1]. In addition to their main function as membrane building blocks, phospholipids have also been shown to be involved in intracellular trafficking and signal transduction, thus having a more dynamic role in cellular physiology [2]. A type of low-abundant phospholipids, called phosphoinositides (PIs), in addition to being a component of the cell membranes, interact with numerous effector proteins and were shown to serve as either signaling molecules themselves or to generate secondary messengers within different cells [3]. To date, PIs have been shown to serve important functions in processes such as cytoskeleton reorganization and membrane curvature generation upon endo-, exo-, or phagocytosis [4,5], polarized cell migration [6,7], cell adhesion [5,8,9], cellular transport utilizing either ion channels [5], concentration gradient, or protein and lipid transfer proteins, as well as receptor-mediated signaling [10,11,12] and gene expression [13]. Despite the range of different PI-dependent cellular processes, the extending spectra of evidence confirming the role of PIs in various physiological and pathophysiological states still manage both to surprise and excite the scientific community. PI metabolism is strictly spatially and temporally controlled by a pool of different kinases, phosphatases, and phospholipases, maintaining their levels and determining multiple aspects of cellular fate [8]. A recent mathematical model predicts that there are 19 kinases and 35 phosphatases involved in the PI pathway alone [14,15]. The distorted homeostasis in PI metabolism was shown to be involved in neurodegeneration [11,16] and neuroinflammation [17], oncogenesis [4,18], infection [19,20], and immune response [21,22]. In this review, we highlight the localization, function, and clinical relevance of the two most abundant PIs, phosphatidylinositol-4-monophosphate (PI4P) and phosphatidylinositol-4,5-bisphosphate [PI(4,5)P_2_] (Figure 1), as well as the kinases involved in their main synthesis pathways. We also provide a summary of known tools for the detection of those PIs.

### The (re)Birth of PI4P and PI(4,5)P_2_

**Figure 2:**
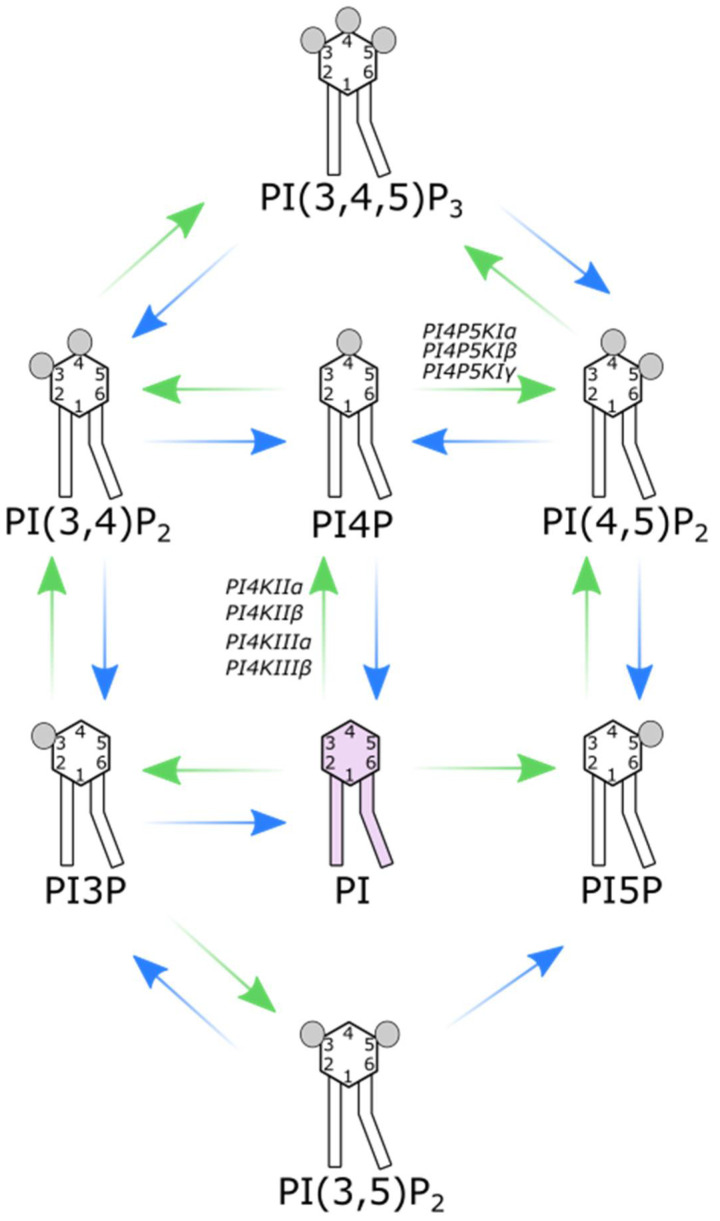
Types of phosphoinositides. There are seven different phosphoinositides depending on the positions of phosphorylation of the inositol ring. Their production is controlled by kinases (green arrows) and phosphatases (blue arrows).

The parent molecule for PI synthesis is phosphatidylinositol (PtdIns), which consists of a myo-inositol ring linked by a phosphodiester bond to a diacylglycerol (DAG) backbone with two hydrophobic fatty acyl chains [19]. PtdIns is synthesized at the cytoplasmic leaflet of the endoplasmic reticulum (ER), but it can later either be flipped to the luminal leaflet and form glycosylphosphatidylinositol-linked proteins (GPIs) or redistributed across other membranes by vesicular transport or lipid transport proteins (LTPs) to generate other PIs [12,23]. Due to the fact of its steric properties, the inositol ring is available for phosphorylation at three of its hydroxyl groups (3, 4, or 5-OH) (Figure 1). Depending on the degree and position of phosphorylation, the inositol ring yields a total of seven different PIs (Figure 2). The number and site of the phosphorylation change the extent of their negative charge and modify their steric properties, both of which influence their distinctive binding affinities [24]. Interestingly, one distinguishing characteristic of mammalian PIs is their DAG composition, mostly enriched in polyunsaturated stearic (sn-1) and arachidonic acid (sn-2). Other phospholipids were not shown to be enriched in one specific fatty-acyl chain combination, which suggests that the PIs are being recycled [25]. The phosphorylation of PIs is a reversible process, meaning their levels are tightly controlled by multiple kinases and phosphatases [24] (Figure 2). Consequently, both PI4P and PI(4,5)P_2_ can be generated by three distinct pathways. PI4P mainly originates from the phosphorylation of PtdIn on the 4-OH by the action of phosphatidylinositol-4-kinases (PI4Ks) [26]. Two minor routes involve either dephosphorylation of PI(4,5)P_2_ on 5-OH by 5-phosphatases (OCRL, synaptojanin1/2, INPP5) or dephosphorylation of PI(3,4)P_2_ on 3-OH by 3-phosphatase PTEN [11,27]. PI4P can be further phosphorylated on 5-OH by type I phosphatidylinositol-4-phosphate 5-kinases (PI4P5KI), presenting a main route of PI(4,5)P_2_ formation. Alternative routes include PI5P phosphorylation on 4-OH by type II phosphatidylinositol-5-phosphate 4-kinases (PI5P4KII) or PI(3,4,5)P_3_ dephosphorylation on 3-OH by 3-phosphatases PTEN and TPIP [24,27]. Different isoforms of these enzymes reside in specific subcellular compartments, thus forming localized pools of their products [20]. We further discuss the structure, localization, and contribution to PI4P and PI(4,5)P_2_ pools, biological and clinical relevance, and finally the pharmacological potential of different PI4K and PI4P5KI isoforms, while phosphatases are reviewed elsewhere [27,28,29].

## 2. PI4Ks

PI4Ks are present as four different isozymes divided into two subfamilies: type II kinases (PI4KIIα and PI4KIIβ) and type III kinases (PI4Kα and PI4Kβ) [20]. The nomenclature lacks type I variants since they were later shown to be phosphatidylinositol-3-kinases (PI3Ks) [5]. The catalytic domain of type III PI4Ks is similar to those of PI3Ks, possibly having a role in interactions with other proteins [30,31]. Surprisingly, the structure of type II kinases varies significantly from other lipid kinases, resembling those of protein kinases [32]. Thus, the two types have distinct stimulation/inhibition routes [19]. For example, type II kinases are sensitive to adenosine and Ca^2+^ inhibition and insensitive to wortmannin (pan-anti-PI3K), while type III show the opposite susceptibility [33]. Furthermore, the smaller type II kinases mostly act as monomers [34] and are destined for membrane tethering due to the inserted palmitoylation sites [33]. In contrast, larger type III kinases have a dimerization region [34], are cytosolic, and shuffle among membranes of different cellular compartments or transiently associate with the PM [19].

### 2.1. PI4KIIα

PI4KIIα (55 kDa) is considered to be the most abundant and active isoform in mammalian cells, generating almost half of the total cellular PI4P levels [20,35]. PI4KIIα is constitutively incorporated within the membrane, possibly due to the distinctive highly hydrophobic pockets in addition to palmitoylation, thus acting as an integral membrane protein [36,37]. PI4KIIα is palmitoylated in the Golgi apparatus, and since the palmitoyltransferases require cholesterol, both PI4KIIα membrane association and activity depend on local cholesterol levels [38,39]. PI4KIIα is known to be recruited to the PM by Rac1 [37]. The structures of PI4KIIα and PI4KIIβ show a high level of similarity in their C-terminal but lower in their N-terminal regions [40]. PI4KIIα and PI4KIIβ share an N-terminal palmitoylation site, but only PI4KIIα has a clathrin adapter protein-3 (AP-3) binding site in the N-terminal proline-rich region [33,41], which is required for transport from late endosomes to lysosomes [39]. In addition to the greater trans-Golgi network (TGN) and endosomal PI4P pools, PI4KIIα also contributes to sub-pools in the PM, lysosomes [42], and multiple small post-Golgi vesicles [38]. It was shown to be associated with various cellular processes, such as autophagy [43,44,45], lysosomal delivery [41], lysosomal repair [46], endosomal receptor sorting [20], exocytosis [47], signal transduction [48,49], actin remodeling [47], and sphingomyelin synthesis [50].

Due to the fact of its abundance in mammalian cells, its role in biological processes and clinical perspective is of much interest. The role of PI4KIIα in oncogenesis is so far the most researched one, with multiple studies supposing its role as an oncoprotein, overexpressed in several different cancer types [38]. The loss of PI4KIIα was shown to be involved in tumor cell apoptosis by driving endosomal EGFR degradation [51] leading to impaired antiapoptotic Akt signaling [52], which makes PI4KIIα an interesting therapeutic target in EGFR-dependent tumors such as breast cancer [51], glioblastomas, and some subtypes of lung and colorectal cancers [53]. It was shown that the complex formed by PI4KIIα and AP-3 regulates lysosomal function in healthy cells, but forming a complex with RNA-dependent protein kinase R (PKR) promotes misfolded prion protein clearance and viability in cancerous cells [42]. Since the expression of cellular prion proteins is known to promote cancer proliferation and metastasis [54], targeting their accumulation via PI4KIIα complex destabilization could be an interesting therapeutic target. Another approach could be the disabling of angiogenesis in cancer cells by downregulating PI4KIIα, which was shown to inhibit human epidermal growth factor receptor 2 (HER-2) activity and lead to a decrease in hypoxia-inducible factor 1α (HIF1α) overexpression, as well as disruption of PI3K-mediated increase in pro-angiogenesis factors [49].

Although both type II kinases are ubiquitously expressed, PI4KIIα was shown to have higher expression in synaptic vesicles [33,55], posing the question of its role in neurological defects. Interestingly, PI4KIIα binding partner AP-3 is known to regulate endosomal cargo transport to axons and synaptic vesicles, as well as synaptic vesicle formation. It is yet to be investigated if this interaction is involved in progressive motor disabilities seen in PI4KIIα-deficient mice, a phenotype resembling human hereditary spastic paraplegia [56]. In addition, it was shown that the N-terminus of PI4KIIα is phosphorylated by glycogen synthase kinase 3 (GSK3), which is known to be essential for neurodevelopment and CNS function. The phosphorylation enables the binding of the kinase to AP-3, mediating neuronal receptor trafficking and expression [57]. Furthermore, PI4KIIα-mediated PI metabolism was also shown to be an interesting target in Alzheimer’s disease treatment by modulating γ-secretase activity, an enzyme responsible for amyloid β-peptide processing [58]. Recently, patients with biallelic deficiency of the enzyme were shown to suffer from severe encephalopathy and movement disorder, possibly due to Rab7-associated late endosome-lysosome trafficking defects [59]. Another intriguing case is of a patient diagnosed with *cutis laxa*, a severe connective-tissue disorder with common neurological symptoms, bearing a PI4KIIα mutation, for the first time posing the question of the importance of lipid metabolism in the pathophysiology of this disorder [60].

In addition to the rising evidence of its role in oncogenesis and neurological defects, there are reported functions of PI4KIIα in metabolic disorders, such as diabetes and Gaucher disease as well [61,62]. In diabetes, it was shown that PI4KIIα is involved in mediating exocytic insulin release by regulating protein kinase D (PKD) activity [61]. In Gaucher disease, PI4KIIα depletion leads to the failed transport of lysosomal integral membrane protein type 2 (LIMP-2) and β-glucocerebrosidase (GBA) enzyme secretion [62]. PI4KIIα was also shown to be important in *Chlamydia* species bacterial infection by mediating replication complex formation through ADP-ribosylation factor 1 (Arf-1) binding [35]. Finally, PI4KIIα was shown to form a tri-component complex with AP-3 and BLOC-1, both showing defects in Hermansky–Pudlak syndrome characterized by albinism and impaired platelet aggregation due to defects in endosomal sorting [63,64]. In addition, the role of PI metabolism in maintaining hemostatic function has been shown in human endothelial cells in which the PI4KIIα depletion caused the abnormal length of Weibel–Palade bodies and impaired folding of von Willebrand factor [65], an important component of primary platelet adhesion during vessel wall injury.

Altogether, the variety of PI4KIIα-related disorders, especially its supposed role as an EGFR-linked oncoprotein, made it an attractive target for the development of pharmacological inhibitors that could exceed the limitation of previously used nonselective inhibitors such as phenylarsine oxide (PAO) [35,66]. To date, there is one selective, reversible, and commercially available substrate-competitive PI4KIIα inhibitor, PI-273, shown to inhibit breast cancer cell growth [48]. Detailed information on PI-273 and other inhibitors is given in Table 1 (IC50, off-targets, research area, and state of development).

### 2.2. PI4KIIβ

PI4KIIβ (55 kDa) is the less studied and less active type II PI4K [20,35]. Unlike constitutively membrane-bound PI4KIIα, PI4KIIβ is mostly cytosolic or to a smaller extent associated with the PM, TGN, ER, and clathrin-coated vesicles (CCVs) [37,39]. Cytosolic PI4KIIβ is nonpalmitoylated, nonactive, and stabilized by the interaction with heat shock protein 90 (Hsp90) on its C-terminal site [33,36]. Upon platelet-derived growth factor (PDGF)-mediated cell activation, PI4KIIβ is recruited to the PM by Rac1 GTPase and this interaction is responsible for its activation as well. The membrane association potentiates its activity thus making it as active as PI4KIIα. PI4KIIβ has a different hydrophobic pocket structure, thus acting as a peripheral protein [36,37]. In addition, instead of the AP-3 binding site, PI4KIIβ has a binding site for clathrin adapter protein-1 (AP-1) on the N-terminal proline-rich region [33], important for trafficking from TGN to endosomes [47]. PI4KIIβ synthesizes PI4P pools mostly between the TGN and endosomes, as well as sub-pools in late, recycling, or enlarged early endosomes [47,88]. It is associated with vesicular cargo sorting, endosomal receptor trafficking [88], intracellular signaling, phagocytosis [89], and actin remodeling [47]. The role of PI4KIIβ in cancerogenesis is less investigated, but recent findings propose both its antioncogenic and prooncogenic effects. PI4KIIβ was shown to impair apoptosis in carcinoma cells by interaction with the tumor suppressor prostate apoptosis response-4 (PAR-4) [38]. In addition, the interaction of PI4KIIβ and AP-1 is required for Wnt receptor endosomal sorting, a pathway that was seen to be disturbed in human cancerogenesis [88]. In addition, PI4KIIβ was found to be downregulated in human tumor cells, which increases invadopodia formation and promotes cancer metastasis pointing to its antioncogenetic effect [47]. Another interesting role of PI4KIIβ is shown in inflammation, as observed by its upregulation during lipopolysaccharide (LPS) signaling. It was shown that its palmitoylation is a crucial step in the LPS-mediated production of proinflammatory cytokines [90]. In addition, PI4KIIβ was shown to be important in immune response mediating early T-cell activation through CD3 TCR [20]. Interestingly, PI4KIIβ is the more abundant type II isoform in the liver, but the reason for that remains to be elucidated [89]. There is no selective inhibitor for PI4KIIβ reported to date [33]; however, due to the presence of emerging evidence on the role of PI4Ks in a variety of different cellular processes, its crystal structure was recently solved and studies are underway to find selective inhibitors [91].

### 2.3. PI4Kα

Type III PI4Kα (230 kDa) has a nuclear localization sequence (NLS) and a pleckstrin homology domain (PH), possibly allowing its membrane interaction [33,89]. It is mainly located in the ER, transiently associates with the PM, and can also be found at early cis-Golgi compartments, nucleolus [92], multivesicular bodies (MVBs), and outer mitochondrial membrane [35]. It was shown to generate the largest hormone-sensitive PM PI4P pool, even though it is not bound to the membrane itself [89,93]. PI4Kα was shown to be transiently transported and held to the PM by a carrier complex [26]. This complex contains TTC7A (tetratricopeptide repeat domain 7A) for targeting to the PM, EFR3 for tethering, and FAM126A for stabilization [11,94]. The role of PI4Kα in the ER is somewhat less clear. PI4P produced by PI4Kα at the ER could be transported to the PM or the cis-Golgi. An unexpected but likely role of PI4Kα at the Golgi apparatus is the control of coat protein complex I (COPI) trafficking of protein and lipid cargo by altering PI4P levels and association with Rab1 [35]. PI4Kα can also be found in the nucleus where its PI4P pool is likely, at least in part, used for nuclear PI(4,5)P_2_ production [35].

PI4Kα was found to be a crucial component in hepatitis C virus (HCV) replication in the liver. It is recruited by NS5A viral protein to provide PI4P, needed for membranous web formation and its integrity preservation [19,39]. These findings resulted in the search for PI4Kα inhibitors in the hope of novel HCV antiviral therapy [20]. Interestingly, it was reported that an anti-HCV agent simeprevir, could make brain and breast tumor cells more prone to radiosensitization and delay their growth by downregulating antiprogrammed death-ligand 1 (PD-L1) and Akt signaling upon PI4Kα inhibition [95]. Further, PI4Kα loss of function was shown to result in impaired hematopoietic differentiation of myeloid and erythroid lineage through Akt and Erk signaling. It was found that in myeloid and erythroid lineage-linked leukemias, normal PI4Kα signaling is inhibited by upregulation of PI4KAP2, formerly considered a nonfunctional PI4Kα pseudogene [96]. In addition, PI4KA tethering to the PM by EFR3 was found to increase the levels of KRAS, the most common human oncogene at the PM and its downstream signaling, thus providing a novel intervention route in KRAS-dependent carcinomas such as pancreatic, colorectal, or lung carcinoma [97]. Finally, since PI4Kα was already shown to be upregulated in pancreatic cancer cell lines and it was linked with poor prognosis in hepatocellular carcinoma [98], it has been highlighted as an interesting anticancer agent.

PI4KA is highly expressed in the brain, and its defect was found to be associated with neurological phenotypes. PI4KA is thought to be important in early brain development since human fetuses carrying an inactivating mutation had fatal brain abnormalities [99]. It was also shown that the mice with PI4KA deletion in Schwann cells show symptoms of neuropathy, due to the fact of its distorted PI metabolism and actin reorganization, leading to impaired myelination [92]. In addition, hypomyelination was also observed when the PI4KA contact with the PM was destabilized by FAM126A depletion in oligodendrocytes [74,100]. Recently, patients with severe neurodevelopmental issues due to the fact of hypomyelination have been reported to carry the biallelic PI4KA variant [100]. Furthermore, an SNP in the promoter region of PI4Kα was associated with schizophrenia phenotype [19].

Interestingly, there was also a reported role of PI4Kα in intestines, its loss caused fatal intestinal lesions in mice, a phenotype previously seen in patients lacking TTC7A, a protein that targets PI4Kα to the membrane [94]. The intriguing data on the impact of PI4Kα complex partners emphasize the role of adequate PI4Kα targeting to the PM in health and disease. Since PI4Kα is an essential gene, its genetical targeting leads to early embryonic lethality [26,34,68]; therefore, its targeting switched a course to pharmacological inhibition. Nevertheless, a careful design of studies using PI4KA pharmacological inhibitors is needed, since it was shown that sometimes a high dose of PI4KA inhibition can lead to sudden death in mice due to the fact of cardiovascular collapse [68]. To date, there are two potent selective PI4Kα inhibitors reported, GSK-A1 and GSK-F1 [15,33,68,69] (detailed information provided in Table 1).

### 2.4. PI4Kβ

Type III PI4Kβ (92 kDa), in contrast to PI4Kα, lacks a PH domain but instead has a serine-rich region prone to protein kinase D (PKD) phosphorylation and NCS-1 activity-modulating binding site, as well as Rab11 binding site [33]. Along with PI4KIIα and PI4KIIβ, PI4Kβ is another PI4K isoform mainly generating PI4P at the Golgi apparatus [33]. It also greatly contributes to the nuclear PI4P pool and generates PI4P sub-pools in the ER, lysosomal, and outer mitochondrial membrane [35]. Its recruitment to the Golgi apparatus is regulated by Arf1 and ACBD3, and its activity is potentiated by PKD phosphorylation and then stabilized through interaction with the 14-3-3 protein [19,27]. PI4Kβ was shown to be important in regulating structural and functional aspects of the Golgi apparatus, vesicular fusion, cargo sorting, and endocytosis [35]. Like the other PI4K isoforms, PI4Kβ was also shown to be involved in cancerogenesis. It is upregulated in various malignancies such as skin (basal cell carcinoma), brain, as well as breast cancers where it increases Akt signaling through PI(3,4,5)P_3_ generation. Surprisingly, it was shown that this action is due to the fact of PI4Kβ direct binding of Rab11 and recruitment to recycling endosomes, independent of kinase activity [101]. It is also considered to be a potential antimalarial and antiviral therapeutic [33]. PI4Kβ inhibition was shown to impair poliovirus, picornavirus, coxsackievirus replication, and HCV secretion [33], as well as SARS-Cov entry [102]. Upon enteroviral infection, PI4Kβ is recruited by Arf-1, which is activated by Golgi brefeldin A resistant guanine nucleotide exchange factor 1 (GBF-1) interacting with a viral protein 3A. Another mechanism of PI4Kβ recruitment is utilized by the Aichi virus, whose 3A protein interacts with the ABCD3 kinase mobilizing protein [39]. PI4Kβ is a positive regulator of Hedgehog signaling, thus it is important in vertebrate embryonic development and tissue homeostasis and regeneration in adults [103]. Additionally, its inhibition leads to a suppressed immunological response to organ transplants in vivo [33]. Surprisingly, it is involved in Gaucher disease, where it controls the exit of β-glucocerebrosidase from the TGN [39]. Several potent selective PI4Kβ inhibitors are available, including IN-9, IN-10, T-00127-HEV1, BF738735, and enviroxime [15,33] (Table 1). Enviroxime and *Plasmodium falciparum* selective PI4KIIIβ inhibitor MMV390048 are the only PI4K inhibitors so far that entered the clinical studies but were eventually both discontinued or terminated, respectively [15].

It is worth noting that in the same compartments (such as the Golgi apparatus or the PM) different kinases produce the same product, e.g., PI4P. However, it is still not entirely clear why different kinases are performing the same reaction, if and how they work together. Interestingly, the kinases that produce PI4P (types II and III) are evolutionarily distinct from each other [34]. It is taught that since PI4P has many different roles in the cell (e.g., regulating anterograde transport from the Golgi apparatus or regulating lipid transport), this may explain why PI4P-producing kinases have evolved multiple times. Moreover, not all PI4P-generating kinases that produce PI4P at a given organelle are constantly present at that organelle. As explained above, PI4KIIα is constitutively incorporated into the membrane, whereas PI4KIIβ is mostly cytosolic and is recruited to the PM upon cell activation. Therefore, it is possible that the activation state of the cell or its requirements activate different kinases through numerous cell signaling pathways.

## 3. PI4P5KIs

PI4P5KIs present a major PI(4,5)P_2_ synthesis pathway due to the fact of significantly higher levels of available PI4P in contrast to PI5P [8]. Interestingly, even though PI4P5KIs preferably bind PI4P, they are not substrate-exclusive like PI4Ks. PI4P5KIs were also shown to bind PI3P or PI(3,4)P_2_, phosphorylate these substrates on 5-OH, and finally lead to PI(3,4,5)P_3_ generation [8,40]. PI4P5KIs are present in mammalian cells in three different isoforms: α, β, and γ [55]. PI4P5KIs show a high level of structural similarity in the core kinase domain (70–80%), mostly differ in the C-terminal domain and, to a lesser extent, in N-terminal domains or a kinase insert, altering their localization and interaction preference [3,40,87]. A great deal of its interaction partners can also bind PI(4,5)P_2_, modulating their function [104]. It is considered that the activation loop insert is responsible for sensing local PM levels of phosphatidylserine and cholesterol thus targeting PI4P5KIs to the PM and controlling their activity [12]. PI4P5KIs activity was also shown to be modulated by phosphatidic acid, small GTPases (Rac and Arf) [105], phosphorylation, or Wnt signaling [55], and suggested being mediated by homo- or hetero-dimers formation [12]. PI4P5KIs can all be found in the PM to a different extent, and some of them localize in the Golgi apparatus, nucleus, and endosomal compartments as well. They are known to be involved in various cellular processes such as endocytosis, exocytosis, apoptosis, and regulation of ion channels [20,40], but they are mostly known for their role in actin reorganization [106]. PI4P5KIs have been shown to be involved in multiple aspects of breast, pancreatic, and colorectal cancer oncogenesis [87].

### 3.1. PI4P5KIα

PI4P5KIα (68 kDa) was shown to mainly localize in the inner leaflet of the PM and the Golgi apparatus but was also found on the site of membrane ruffling and in nuclear speckles [8]. It was shown that the G protein-coupled receptor (GPCR) stimulation translocates the kinase across the membrane and potentiates its activity. PI4P5KIα was shown to regulate phagocytic microbe ingestion by creating a PI(4,5)P_2_ pool which facilitates WASP and ARP2/3-mediated actin reorganization [3,20]. For example, it was shown to facilitate actin reorganization in T cells, thus allowing HIV-1 entry [20]. Interestingly, it was suggested that its activity in the nucleus can be potentiated as a response to oxidative stress [8]. PI4P5KIα is upregulated in breast and prostate cancer cells, thus leading to poorer outcomes in KRAS/Akt- and p53-dependent oncogenesis [107]. So far the only known PI4P5KIα inhibitor, ISA-2011B, is considered to be a promising anticancer agent in advanced prostate cancer and triple-negative breast cancer [38] (Table 1).

### 3.2. PI4P5KIβ

PI4P5KIβ (68 kDa) is mostly found in the perinuclear region [3], vesicular structures, and to a lesser extent in the PM [5]. It was shown that the PI4P5KIβ interaction with Rac-1 GTPase regulates both its PM localization and activity. It forms a heterodimer with PI4P5KIα, which can compensate for its kinase activity if needed [20]. PI4P5KIβ was shown to be involved in various aspects of actin dynamics during cell migration and activation. It is targeted to the leading edge of the cell, where its product PI(4,5)P_2_ induces actin reorganization through N-WASP activation [8]. It was shown that the expression of PI4P5KIβ is needed for actin polymerization by mediating comet formation and elevating stress fibres formation [19]. In addition, the inactive form leads to disturbed EGFR-mediated endocytosis, responsible for clathrin and dynamin trafficking to the PM [40]. Loss of PI4P5KIβ was shown to have a major impact in mediating platelet aggregation by lowering the levels of inositol 1,4,5-triphosphate (IP3), a product of PI(4,5)P_2_ cleavage, whose generation represents one of the first steps in platelet activation [108].

### 3.3. PI4P5KIγ

PI4P5KIγ (~90 kDa) is considered to be the major source of PI(4,5)P_2_ at the PM [12]. Human PI4P5KIγ is a highly diverse family consisting of six splice variants, differing in C-terminal length and all having specific subcellular localizations and interaction partners [3,20]. PI4P5KIγ-v1 represents the most abundant splice variant in most tissues. It is mostly found in the PM, where it is involved in phagocytosis [20]. It was shown to be involved in the process of microbial attachment during macrophage phagocytosis by elevating actin depolymerization. PI4P5KIγ-v2 was shown to be the second most abundant isoform, the one mostly expressed in the brain. It mostly generates the PM PI(4,5)P_2_ pool but can be found in focal adhesion sites as well. Its role was observed in multiple aspects of cell migration. It is considered to be important for focal adhesions assembly by PI(4,5)P_2_ generation, focal adhesion kinase (FAK) activation, and interaction with talin and integrin receptors [3,4,19]. PI4P5KIγ-v2 also maintains cell polarity, another aspect of cell migration by promoting E-cadherin transport to the site of adherens junctions assembly. The cell-cell contact sites based on E-cadherin are an important component of epithelial cancer metastasis. Interestingly, PI4P5KIγ expression was linked to poor prognosis in breast cancer by regulating crucial steps in cancer metastasis, E-cadherin cell-cell contacts, and EGFR-stimulated cell migration [109]. The interaction of PI4P5KIγ-v2 with talin or clathrin adaptor AP2 was also shown to regulate endocytosis of synaptic vesicles, thus mediating neuronal activation. PI4P5Kγ-v3 was observed in the PM but only in neuronal cells across multiple brain regions [20]. PI4P5Kγ-v4 is found in nuclear speckles and PI4P5Kγ-v5 in the PM and endosomal compartments [3,19]. PI4P5Kγ-v5 is needed in endosomal EGFR sorting for lysosomal degradation. One of the mechanisms involves the interaction of PI4P5Kγ-v5 and its product PI(4,5)P_2_ with sorting nexin 5 (SNX5) leading to EGFR sorting and lysosomal degradation. On the contrary, the same interaction with SNX5 can prevent E-cadherin degradation [110]. PI4P5Kγ-v5 was also shown to have an important role in modulating endosomal maturation. It is needed for Rab7a recruitment to early endosomes, a step that is crucial for Rab5a to Rab7a replacement required for the maturation of early endosomes to late endosomes [104]. Finally, PI4P5Kγ-v6 is mostly found in the PM [111].

PI4P5KIγ was shown to be an essential enzyme during embryonic development since its depletion is lethal in mice [112,113]. The variant was shown to be enriched in the brain, its deficiency leading to neurotransmission defects. It was reported that a mutation in PI4P5KIγ leads to neonatally lethal congenital contracture syndrome type 3 (LCCS3), characterized by motoric neuron degeneration possibly due to the fact of PI(4,5)P_2_ deficiency in the brain [3,114,115]. Its loss disables the cytoskeleton linkage with the PM in platelets and their precursor cells megakaryocytes [19,116]. PI4P5KIγ is upregulated in colorectal cancer cells, being responsible for cell proliferation and growth through Akt signaling. In this study, the UNC3230 inhibitor was used [87], but it was later shown to lack selectivity. However, due to the lack of a more sensitive inhibitor, it is still widely used (Table 1).

## 4. PI4P: A Huge Burden on a Small Lipid

**Figure 3:**
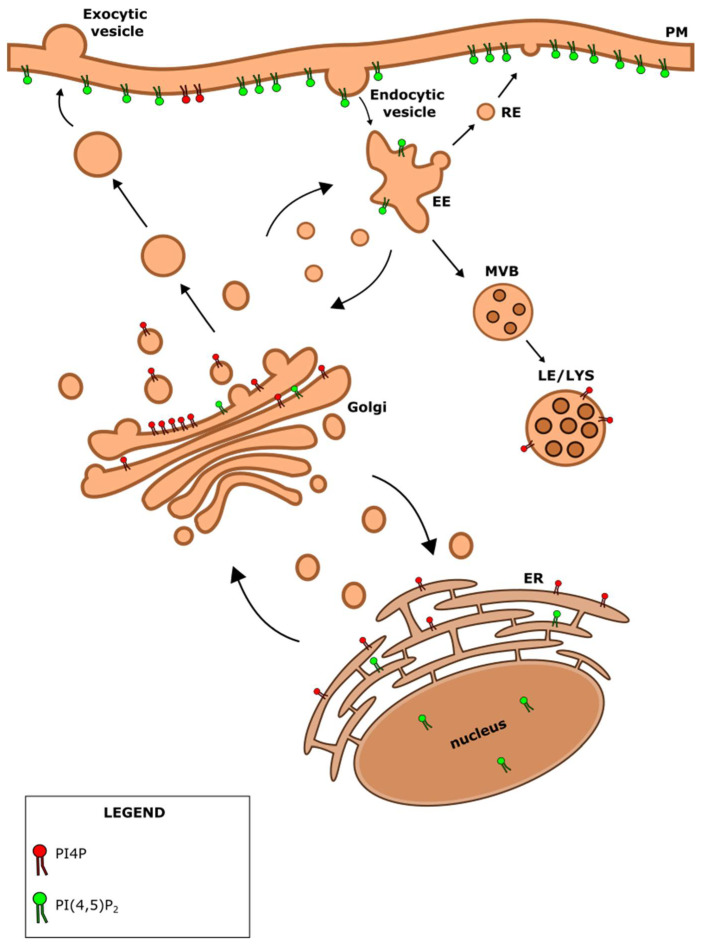
Phosphoinositides in the cell. PI4P (in red) is localized at the Golgi apparatus, endoplasmic reticulum (ER), plasma membrane (PM) and late endosomes/lysosomes (LE/LYS). PI(4,5)P_2_ (in green) is localized at the PM, early endosomes (EE), ER and the nucleus. RE-recycling endosomes, MVB-multivesicular body.

PI4P is mostly localized at the Golgi apparatus and the PM, but it can also be found in the ER [117] and cytoplasmic vesicles such as late endosomes and lysosomes [118] (Figure 3). The late endosomes/lysosomes localization was shown only recently with a novel PI4P probe that utilizes the P4M domain of SidM protein from *Legionella pneumophila* [118]. SidM is localized to bacteria-containing vacuoles where ER-derived materials are recruited through binding to PI4P.

At the Golgi apparatus, PI4P binds effector proteins and regulates the anterograde transport from the Golgi apparatus to the PM via the TGN [119,120,121], impacts protein targeting to endosomes, vesicle formation, and maintenance of Golgi resident enzymes [39,119,120,121]. On the PM it can serve as a regulator of the PM stability and PI(4,5)P_2_ [122] levels, as well as regulate ion channels [39,122]_._

### 4.1. PI4P at the Golgi Apparatus: Glycosylation and Anterograde Trafficking

The Golgi apparatus consists of *cis*-, *medial*-, and *trans*-Golgi cisternae and PI4P is mostly localized and the TGN [123]. It is believed that this is the case because Sac1, the enzyme that dephosphorylates PI4P, is mostly localized to the *cis*- and *medial*-Golgi. Not only that PI4P depletion causes a defect in the Golgi function, but abnormal levels of PI4P also lead to a defective Golgi and its enzymes. Sac1 knockdown (KD) by RNAi causes the PI4P levels to rise at the *cis*- and *medial*-Golgi. The Golgi becomes bigger and fragmented, PI4P is mislocalized at punctate and peripheral structures, cell proliferation is decreased, and mannosidase II and N-acetylglucosamine transferase-1 are translocated from the Golgi to other intracellular and periphery membranes. In addition, Sac1 KD selectively alters *N*- and *O*-glycosylation. Specifically, it decreases the addition of poly-N-acetylgalactosamine repeats on complex, multi-antennary N-glycans and reduces levels of Galβ1-3GalNacα-O-Ser/Thr. It was hypothesized that the exclusion of PI4P from the Golgi cisternae may be essential for preventing anterograde passage of Golgi enzymes to the cell periphery or that COP-1-mediated retrograde transport of Golgi enzymes may be impaired leading to their peripheral accumulation [123].

At the Golgi apparatus, PI4P recruits clathrin adaptors and Golgi-localized, Gamma-adaptin ear homology, Arf-binding proteins (GGAs), critical for lipid transport and cargo delivery from the Golgi apparatus to the PM [124]. Depletion of PI4P at the Golgi apparatus leads to a decline of the vesicular trafficking from the Golgi apparatus to the PM, almost completely shuts down the trafficking from the Golgi apparatus to the MVBs, and leads to the dissociation of GGA1 and GGA2 (but not GGA3) from the Golgi apparatus. Interestingly, early studies showed that the Golgi depletion of PI4P impairs PM PI(4,5)P_2_ pool, at least for a minor part. When PI(4,5)P_2_ levels are decreased in the PM, its recovery is slower when the cell is depleted of the Golgi pool of PI4P [124].

### 4.2. PI4P at the Plasma Membrane

More recent findings suggest that PI4P and PI(4,5)P_2_ have independent roles in the integrity and identity of the PM [125]. Hammond et al. reported that the selective depletion of PM pools of PI4P by recruitment of Sac1 to the PM (the rapamycin-inducible dimerization of FK506 binding protein, FKBP and the fragment of mTOR that binds rapamycin, FRB) does not affect the clathrin-mediated endocytosis, the formation of secondary messengers PI(3,4,5)P_3_ and PI(3,4)P_2_ as well as IP_3_ indicating that PI4P does not maintain the functionally relevant pool of PI(4,5)P_2_ at the PM [125]. If not as a precursor of PI(4,5)P_2_, what is the role of PI4P at the PM? Since it has been shown that PI(4,5)P_2_ can regulate the activity of some ion channels [126], it was hypothesized that PI4P could also have a similar role [125]. Indeed, the depletion of PI4P and PI(4,5)P_2_ but not PI4P or PI(4,5)P_2_ alone led to the inhibition of the capsaicin-activated transient receptor potential vanilloid 1 (TRPV1) cation channel [125]. These findings suggest that either lipid is sufficient for the channel activity and that PI4P at the PM has an autonomous contribution to the polyanionic lipid pool that defines the inner leaflet of the PM. This in turn would make PI(4,5)P_2_ free for its other roles.

### 4.3. PI4P at Membrane-Contact Sites

The localization and role of PI4P have also been implied at membrane contact sites (MCSs) [127]. MCSs are regions where membranes of distinct organelles are tethered and in close proximity, where nonvesicular lipid transfer occurs [128]. They have a role in intracellular signaling, lipid exchange and metabolism, and organelle function [128,129]. Lipid exchange is carried out by oxysterol-binding protein (OSBP) and OSBP-related proteins (ORPs) that are enriched at MCSs by binding to PI4P. At ER-Golgi MCSs, the hydrolysis of PI4P directs the exchange of PI4P from the Golgi to the ER and cholesterol from the ER to the Golgi in a four-step process [127]. At ER-PM MCSs, PI4P transfers from the PM to the ER while transferring PS in the opposite direction [117]. It is worth mentioning that the KD of Sac1 decreases the dissociation of PI4P from the PM. It was hypothesized that this implies that PI4P consumption by Sac1 at the ER is necessary for efficient counter-transport by allowing the OSBP-related domains (ORD) to favor PS binding over PI4P binding at the ER [117]. Furthermore, this exchange could help to control the levels of PI4P at the PM and selectively enrich PS in the PM.

## 5. PI(4,5)P_2_: A Dual Role

PI(4,5)P_2_ mainly accumulates at the PM but is also found in low abundance in intracellular membranes such as the ones of the Golgi apparatus, endosomes, ER, and electron-dense structures within the nucleus [130] (Figure 3). At the PM, PI(4,5)P_2_ is shown to be important for maintaining membrane curvature, clathrin-mediated endocytosis, focal adhesion assembly, and regulation of synaptic vesicle recycling [131], processes that include actin reorganization. PI(4,5)P_2_ has been also implicated in various nuclear processes such as RNA processing, nuclear export, regulation of nuclear actin, and chromatin remodeling [35]. In the nucleolus, PI(4,5)P_2_ was shown to colocalize and associate with transcription factor UBF, thus having a role as a transcriptional regulator as well [132]. Furthermore, PI(4,5)P_2_ is a signaling molecule and serves as a source of secondary messengers inositol 1,4,5-trisphosphate (IP_3_) and DAG [19].

### 5.1. PI(4,5)P_2_ in Clathrin-Mediated Endocytosis

CCVs are coordinately assembled at the PM and the polymerization of the outer clathrin layer is assisted by adaptor proteins which bind to clathrin, membrane lipids, and cargo proteins [133]. The central adaptor protein is adaptor complex 2 (AP-2) which binds all the above-mentioned parts needed for the CCV formation, and the most important membrane lipid is PI(4,5)P_2_. It has been shown that elevated levels of PI(4,5)P_2_ lead to the accumulation of CCVs at nerve endings [131] and to the increased levels of endocytosis of transferrin receptors, the association of AP-2 with the membranes, and the number of CCVs at the PM [134]. Adaptor proteins bind to PI(4,5)P_2_ by different domains such as PH, AP180 N-terminal homology (ANTH) domain, or epsin N-terminal homology domain (ENTH) but other proteins can also associate with PI(4,5)P_2_ [133]. These proteins mediate the function of the actin cytoskeleton, like Wiskott-Aldrich syndrome protein (WASP) and profilin. Once a CCV enters the cell, it goes through the endocytic pathway and PI(4,5)P_2_ in CCVs is exchanged for PI3P which allows the formation and maturation of early endosomes and the formation of recycling endosomes [135]. The inability of PI(4,5)P_2_ hydrolysis by the KD of the oculo-cerebro-renal syndrome of Lowe (OCRL) phosphatase results in the accumulation of PI(4,5)P_2_ at the CCVs. When PI(4,5)P_2_ is accumulated on CCVs, enlarged early endosomes form and the neuronal-WASP-dependent increase in endosomal F-actin is induced. Enlarged early endosomes with abundant actin cannot form recycling endosomes and the recycling machinery is shut down [135]. PI(4,5)P_2_ also plays a role in phagocytosis where the levels correlate with the formation of actin during pseudopod extension [19]. It has also been suggested that PI(4,5)P_2_ has a role in exocytosis, but the exact mechanisms yet need to be revealed [136]. However, it is assumed that it could be through the regulation of actin reorganization.

### 5.2. PI(4,5)P_2_ as a Signaling Molecule

The activation of phospholipase C (PLC) leads to PI(4,5)P_2_ hydrolysis to second messengers IP_3_ and DAG [137]. DAG then activates protein kinase C (PKC) which has an important role in several different signaling cascades. IP_3_ is soluble and binds to its receptor on the ER and increases intracellular calcium levels that activate calcium-sensitive signaling molecules. Furthermore, PI(4,5)P_2_ itself can act as a regulator of ion channels [126,138]. PI(4,5)P_2_ in most cases increases channel activity while its hydrolysis by PLC reduces the channel activity. PI(4,5)P_2_ can act on inward-rectifier K^+^ channels, voltage-gated K^+^ channels, voltage-gated Ca^2+^ channels, sensory transduction channels, and others [138].

## 6. Tools for Detection of Intracellular Phosphoinositides

To understand the characteristics and functions of PIs within cells, various tools for their visualization and detection have been developed. The visualization of PIs is usually performed using antibodies and/or genetically encoded probes that contain specific protein domains fused with fluorescent proteins [139]. These allow for the determination of the intracellular localization of PIs, relative levels in different compartments, and changes in their levels or distribution in response to genetic or pharmacological manipulation of PI metabolizing enzymes. Furthermore, in recent years researchers developed mass spectrometry analysis for PI detection. Every method for PI detection has its advantages and limitations, so their selection may vary owing to experimental design. Nevertheless, together they significantly contributed to our understanding of PIs and their specific intracellular roles. Here, we will focus on tools commonly used for the detection of PI4P and PI(4,5)P_2_.

### 6.1. Antibodies

Commercial antibodies are available against almost all PIs, including PI4P and PI(4,5)P_2_, and have been widely used for determining their localization and abundance within the cells. However, the antibody detection of these PIs has several caveats and limitations. One of them is that an antibody is much larger than a PI for which the antibody is supposed to be specific thus making it hard to distinguish between two PIs that differ only in one phosphate group [140]. PIs that are already bound to proteins in cells are not reachable to antibodies, therefore underestimating their amount or localization. In addition, lipids are not subject to formaldehyde fixation thus not being immobilized, and using standard detergents might result in the extraction of lipids [139]. Nevertheless, there are a few reliable commercially available antibodies that have been tested and their specificity proved, among which are antibodies for PI4P and PI(4,5)P_2_. However, antibody detection can provide information about the localization of these lipids only at a given time point and cannot be used to detect transient changes [139]. Next, for the antibody to gain access to intracellular compartments, cells need to be fixed and permeabilized which can lead to differences in the antibody accessibility between different cellular compartments. This has been observed with PI4P and PI(4,5)P_2_ where, although localized at the PM and/or the Golgi apparatus, they cannot be visualized in both at once [122]. Hammond et al. established specific immunocytochemical protocols for the preservation of different membranes and visualization of PM or intracellular PI4P and PI(4,5)P_2_ pools [122]. However, the established staining protocols do not stain confidently all cell types and need further optimization [141]. In addition, PIs show different localization in diverse cell types [141].

### 6.2. Genetically Encoded Probes

Genetically encoded probes contain a specific PI-binding domain fused with fluorescent proteins and are the most valuable tool for the visualization of PIs inside the cells [139]. They are compatible with live cells and allow the analysis of not only the intracellular localization of PIs but also the dynamic changes in their relative levels, as well as the changes in PI levels in response to stimuli. Although shown as useful, several aspects need to be considered when using genetically encoded probes to monitor PIs. The use of protein domains as probes depends on their specificity for the target PI, an affinity that allows the detection of the PI, and an understanding of secondary interactions that may interfere with the distribution of the probe. Some probes can recognize several types of PIs therefore not being selective [142]. Since PIs are bound to endogenous regulatory proteins in cells, their detection by expressed probes depends on the accessibility to those PI pools. Overexpression of probes can sequester their target lipid and, in this way, disturb lipid interaction with downstream effector proteins leading to a dominant negative effect. In addition, probes could be recruited to membranes by interacting with other proteins and in this way sequester protein function [142].

There are several families of PI-binding domains, the largest being the PH domain which is used primarily to monitor the distribution and levels of PI4P and PI(4,5)P_2_ within the cells (Table 2). The PH domain of PLCδ1 was not only useful for the assessment of the PI(4,5)P_2_ localization within the cell but also to determine the nanoscale spatial organization of this PI in the PM using single-molecule superresolution microscopy [143].

The limitation of genetically encoded probes is that their specificity and affinity can vary greatly. For example, pools of PI4P on the Golgi and the PM cannot be visualized using the same PH domain. The Golgi pool of PI4P can be detected with the PH domain of OSBP and FAPP1, whose association with the Golgi membrane requires the small GTPase Arf1 [121,145]. In contrast, the localization of the PH domain from OSH2 is not related to the Arf1 binding so this domain is used to visualize the PM pool of PI4P [144]. The localization of PI4P in the late endosomes/lysosomes, in addition to Golgi and PM, was shown only recently with a novel PI4P probe that utilizes P4M domain from SidM protein. As mentioned above, SidM is a secreted effector protein of *Legionella pneumophila* that binds PI4P [118].

PI(4,5)P_2_ distribution can be visualized using the PH domain from PLCδ1 protein that specifically labels PM pools of this lipid. The PLCδ1-PH domain has been used to monitor PI(4,5)P_2_ hydrolysis and to investigate its role in phagocytosis, calcium-dependent exocytosis, and clathrin-mediated endocytosis [150]. As mentioned earlier, when PLC is activated, it cleaves PI(4,5)P_2_, producing DAG and IP_3_ [151]. This results in the translocation of the PLCδ1-PH-containing probe from the PM to the cytosol, which reveals a decrease in PM PI(4,5)P_2_ levels. However, because PLCδ1-PH binds to the inositol group of PI(4,5)P_2_, it is unclear whether this decrease is a result of a PI(4,5)P_2_ depletion or an IP_3_ increase. Therefore, to confirm the presence of not only PI(4,5)P_2_ but also other PIs and to provide conclusions about their intracellular localization and levels, the use of multiple probes combined with other techniques is recommended.

The Tubby domain is used to detect intracellular PI(4,5)P_2_, but it can also recognize PI(3,4)P_2_ and PI(3,4,5)P_3_ [147]. However, it must be noted that the probe containing the Tubby domain is suitable for visualization of PI(4,5)P_2_ because it is the only PI that is present at sufficient concentration to drive the localization of the probe [11].

Finally, the ENTH domain of the Epsin1 protein was recently developed to overcome the limitations of the PLCδ1-PH and Tubby domains. It has been shown that the ENTH domain binds to PI(4,5)P_2_ without binding to IP_3_, it has a low affinity for PI(4,5)P_2_ and does not interfere with its dynamics if overexpressed [148]. Therefore, it has been shown as a highly sensitive probe able to detect PLCβ-dependent PI(4,5)P_2_ depletion. Furthermore, the overexpression of this probe did not attenuate G_q_PCR signaling (unlike the PLCδ1-PH domain) making it suitable for the detection of minute changes in PI(4,5)P_2_ levels.

### 6.3. Mass Spectrometry

The development of mass spectrometry analyses allowed the growth of a new branch called lipidomics [152], which allowed systems-level analysis of lipids in different human diseases. For the analysis of PIs in the cell, researchers have used fast atom bombarded mass spectrometry (FAB-MS), matrix-assisted laser desorption and ionization/time of flight mass spectrometry (MALDI-TOF-MS), and electrospray ionization mass spectrometry (ESI-MS). The use of ESI-MS was shown to be valuable in the sense it can distinguish acyl chain contents of PIs and it is suitable only for the investigation of high abundant PIs [152], suggesting that all the lipids could not be extracted or identified [153]. Indeed, it was discovered that the extraction method for PIs is quite challenging. However, the optimization of the extraction methods and using tandem mass spectrometry analyses (MS-MS) yielded better results in identifying PIs, although it still could not resolve for spatial isomers, only for the number of phosphate groups [153,154]. Today, using the high-performance ion chromatography-coupled selected reaction monitoring mass spectrometry (IC-MS/MS) it is possible to resolve PIs positional isomers for their quantification in both tissues and cells [155]. Using this method, it has been shown in human platelets that upon activation with the collagen-related peptide the levels of PI4P and PI(4,5)P_2_ significantly increase implicating that both PIs have an important role in platelet activation [155]. These, and other new methods that will be developed in the future will allow for a detailed analysis of the structure, localization, and function of all PIs.

## 7. Conclusions

The numerous localizations and roles of PI4P and PI(4,5)P_2_ in the cell clearly show that they are irreplaceable and valuable components of the cell membranes. The fact that mutations in the kinases that produce PI4P and PI(4,5)P_2_ result in many diseases enhances the importance of these lipids in both physiology and pathology. This drives the development of new and improved methods for their visualization and quantification that aim to clarify the exact localization and function of PI4P and PI(4,5)P_2_ with their kinases, which is of great importance for the identification of target molecules and the development of new drugs.

## Figures and Tables

**Table 1:** Inhibitors of type II, type III PI4Ks, and PI4P5KIs.

Target Enzyme	Inhibitor	Target IC50 (nM)	Off-Targets, IC50 (nM)	State of Clinical Development	Research Area	References
PI4KIIα	PI-273	470 [48]	no off-targets, highly selective	in vitro	cancer	[48,67]
				in vivo		[48]
PI4KIIIα	GSK-A1	3.1 [15]	>310 [15]	in vitro	basic	[68,69]
					cancer	[70]
					viral infection	[71]
					inflammation	[72]
					neuronal plasticity	[73]
					myelination	[74]
	GSK-F1	16 [15]	PI4KIIIβ, PI3Ks >1600 [15]	in vitro	cancer	[75]
				in vivo	basic	[68]
PI4KIIIβ	IN-9	7 [76]	PI4KIIIα, PI3Ks >150 [76]	in vitro	cancer	[67]
					inflammation	[72]
	IN-10	3.6 [15]	PI4KIIIα, PI3Ks >720 [15,33]	in vitro	inflammation	[72]
	T-00127-HEV1	60 [32]	PI4KIIIα, PIK3CD 10 000 [32]	in vitro	viral infection	[77,78,79]
				in vivo		[78]
	BF738735	5.7 [32]	PI4KIIIα 1700 [33]	in vitro	viral infection	[79,80]
	Enviroxime	120 [32]	PI4KIIIα 1400 [32]	discontinued in phase II clinical trials	viral infection	[15,81]
*Plasmodium* *falciparum* PI4KIIIβ	MMV390048	28 [82]	no off-targets, highly selective	terminated phase II clinical trials	parasitic infection	[15,83]
PI4P5KIα	ISA-2011B	n.d. [15]	p110α n.d. [15]	in vitro	inflammation	[84]
					cancer	[85,86]
				in vivo	cancer	[85,86]
PI4P5KIγ	UNC3230	51 (K_d_) [15]	PI5P4Kγ 4 (K_d_) [15]	in vitro	cancer	[87]

**Table 2:** Phosphoinositide-binding protein domains commonly used for the visualization of PI4P and PI(4,5)P_2_ within cells.

PI	Protein Domain	Intracellular Localization
PI4P	OSH2-PH [144]	PM
	OSBP-PH [145]	Golgi
	FAPP1-PH [121]	Golgi
	P4M SidM [118]	Golgi, PM, LE/Lys
PI(4,5)P_2_	PLCδ1-PH [146]	PM
	Tubby-PX [147]	PM
	Epsin1-ENTH/AP180-ANTH [148,149]	PM

## Data Availability

Not applicable.
